# Do grandparents compete with or support their grandchildren? In Guatemala, paternal grandmothers may compete, and maternal grandmothers may cooperate

**DOI:** 10.1098/rsos.160069

**Published:** 2016-04-20

**Authors:** Paula Sheppard, Rebecca Sear

**Affiliations:** London School of Hygiene and Tropical Medicine, Keppel Street, London WC1E 7HT, UK

**Keywords:** height, child health, resource competition, cooperative breeding, grandmother, grandfather

## Abstract

Previous research has found that the presence of grandparents, particularly grandmothers, is often positively associated with child survival. Little research has explored the potential mechanisms driving these associations. We use data from rural Guatemala to test whether contact with and direct investment (advice and financial) from grandparents is associated with child health, proxied by height. Our results demonstrate the complexity of family relationships and their influence on child health, suggesting that both cooperative and competitive relationships exist within the family. The clearest evidence we find for grandparental influence is that having a living paternal grandmother tends to be *negatively* associated with child height. By contrast, contact with maternal kin appears broadly to be beneficial for child height, although these relationships are weaker. These patterns are mirrored in maternal body mass index, suggesting grandparental influence acts partly through maternal health. These findings support the hypotheses that, under conditions of limited resources, family relationships may be competitive within the family lineage which shares the same resource base, but cooperative when there are few costs to cooperation. Finally, financial assistance from maternal grandfathers is positively correlated with infant length but negatively with the height of older children, perhaps because the receipt of financial support is an indication of need. The provision of advice shows no associations with child height.

## 1. Introduction

Evolutionary anthropologists expect to see broadly positive effects of grandparents on the health of their grandchildren. The cooperative breeding hypothesis [1] and the grandmother hypothesis for the evolution of menopause [2,3], both of which draw on kin selection theory [4], predict that post-reproductive individuals will direct investment towards existing kin, such as adult children and grandchildren (see also [5]). There is empirical evidence which supports this prediction, and which does suggest that the presence of grandparents is beneficial for their grandchildren [6–8]. However, some of this research suggests that grandparental presence is not always beneficial to children, and a few studies even find that grandchild mortality is higher in the presence of grandparents. This has been attributed to the effects of resource competition within families [7,9,10]. Much of the previous research on this topic, at least in low-income settings, focuses on child survival as the outcome of interest. While this is clearly the most important child outcome, it is a crude measure of child health; the impact of grandparents may be underestimated if this is the only outcome measure used, rather than more sensitive measures of child health.

A handful of studies have tested the influence of grandparents (mainly grandmothers) on other measures of child health. These studies suggest that the presence of grandmothers is often correlated with improved child health, but not always, and sometimes beneficial effects are specific to certain children. In the Gambia, Sear *et al.* [11] found that having a living maternal grandmother was associated with improved child height and weight. In Ethiopia, Gibson & Mace [12] found that having a living maternal grandmother was associated with taller girls, but not boys, and that having a living paternal grandmother was associated with increased height in boys but not girls. In India, Leonetti *et al.* [13] report that Bengali paternal grandmothers resident in the household positively influenced child height but co-residence of Khasi maternal grandmothers only impacted on the height of children born to the youngest daughter of the grandmother. If the grandmother lived with an older daughter, the direction of the association is reversed. Yet Khasi grandmothers exert a positive effect on child height for the children of her older daughters if she is not living with them [13]. Also in India, rural Vadodara, Sharma & Kanani [14] found that children who had grandmothers (of unspecified lineage) present in the household were a little less undernourished than those whose were not.

Evidence for associations between the presence of grandfathers and child health is even scarcer, and reported findings give mixed messages. For example, in the Gambia, grandfathers had inconsistent effects on child height and weight, although there was some evidence that having a living grandfather, either maternal or paternal, was associated with shorter child height than if the grandfather was dead [15]. In Ethiopia, Gibson & Mace [12] found no correlations between grandfathers of either lineage and child height or weight, and these null findings were replicated in Thailand [16].

One factor clouding the results of this previous research may be that measures of grandparental investment were fairly simple indicators of availability, such as survival status or co-residence. Studies which have correlated grandparental availability and child health or survival tend to assume that any positive associations are driven by the provision of help from grandparents to adult children and grandchildren (as is predicted by theory), but few actually test this assumption (but see [12] for an exception).

In high-income contexts more has been done to try and unearth what roles grandparents play by looking at direct measures of investment, e.g. child care or financial support. This is possible because contemporary, large-scale demographic datasets which have collected detailed information on such variables are available [8,17,18]. Such studies often report that grandparents improve child health, usually measured as psychological and emotional wellbeing. Again, there are a few studies where grandparents may be negatively associated with child outcomes, but this may be because grandparents give the most help to children most in need [8,19], although this cannot explain variation by lineage [20]. Unfortunately, such detailed data from low-income settings are limited, although data from hunter–gatherers on time allocation of allocarers [21–23], and of downward intergenerational food transfers [24], do support the prediction that grandparents invest in their grandchildren.

Here we use a detailed, large-scale demographic dataset from a low-income country, Guatemala, which contains information on the provision of three types of support from grandparents to adult children and grandchildren, to test whether contact frequency with, or the provision of financial support or advice from, each grandparent is associated with improved child health. Contact frequency is a more sensitive measure of grandparental investment than simple survival status, as grandparents need to be in contact with kin to provide help. Financial support is a direct measure of material assistance, and indirect evidence has shown that increased financial resources available to co-resident grandmothers improved grandchild nutritional status, when resident grandmothers, but not grandfathers, had access to extra funding through the post-apartheid South African old age pension programme [25]. Advice may also be an important form of direct support which grandparents can provide to their adult children, which may influence grandchild health. For example, in an intervention study in Senegal, grandmothers were given information on maternal health practices during and after pregnancy which they then transferred directly to their daughters (in-law), who in turn were more probably to take up this advice compared with a control group whose mothers (in-law) were not given the intervention [26]. Other studies have provided qualitative evidence that grandmothers are a common source of advice around the perinatal period and for child feeding practices (Malawi: Bezner *et al.* [27]; Nepal: Masvie [28]; Gambia: Thompson & Rahman [29]), and recognizing child illness (Ghana: Douglass & McGadney-Douglass [30]), which may explain why caloric intake was found to be higher in children with grandmothers in one Indian study by Sharma & Kanani [14].

As indicated above, previous work, albeit using grandparental survival status or co-residence, has suggested that not all grandparents are equal when it comes to improving child health or survival. Grandmothers, presumably because of their roles in caring for children and providing advice to mothers and children, tend to be more frequently associated with child outcomes, while grandfathers are typically less often associated with child health or survival (with a few exceptions [31]). In particular, the maternal grandmother has the strongest, most frequent and most often positive effect on child survival and health [6,7]. Paternal grandmothers tend to be less commonly beneficial than maternal grandmothers, although they are sometimes associated with higher child survival. Two historical populations also show evidence of higher grandchild mortality in the presence of paternal grandmothers: Japan [32], and Germany, where mothers-in-law were apparently known as ‘the devil in the house’ [33]. This lineage difference between maternal and paternal grandmothers is often attributed to the indisputable genetic link between women, their daughters and their granddaughters (for all other grandparent–grandchild relationships there is at least one link involving paternity uncertainty).

Paternity uncertainty may help explain why maternal grandmothers are more often beneficial to children than paternal grandmothers, but it does not explain why grandmothers are associated with *higher* child mortality in some societies. The answer here may be resource competition within the family [10]. Under conditions of resource stress, there may be competition within the family for those limited resources, particularly among the lineage within which resources are inherited. This was first suggested as the explanation for higher mortality of female children in the presence of maternal grandmothers in a matrilineal Malawian population [9], and subsequently suggested as the reason for less beneficial/more harmful influences of paternal grandmothers in most of the existing literature, as the majority of extant human societies are patrilineal [7,34]. Resource competition has also been suggested as the cause of some negative associations between the presence of grandfathers and child survival, particularly in strongly patrilineal societies where older men have the highest status, and therefore, the best access to food, within households [35].

We contribute to the gap in research on grandparental influences on child health by examining the impact of each different grandparent on child health (approximated by height) in rural Guatemala, and examining *how* grandparents influence child health. We use child height (standardized for age) as a proxy for health because stunted growth is a common outcome of chronic malnutrition and poor health during childhood [36]. Child weight is likely to reflect short-term nutritional assaults to child health and so may be less revealing when considering the long-term impact of grandparental investments. To investigate the influence of grandparents, and the impact of direct measures of grandparental support, we examined (i) how survival status and contact frequency of individual maternal and paternal grandparents impact on child height, (ii) whether direct measures of grandparental support (advice or financial) are correlated with child height, and (iii) if the different types of grandparental contact and investment are more important for infants' or older children's height.

## 2. Data

Data for the Guatemalan Survey of Family Health (Encuesta Guatemalteca de Salud Familiar—EGSF) were collected from four regional departments in rural Guatemala (Chimaltenango, Totonicapán, Suchitepéquez and Jalapa) between May and October 1995 [37]. These data were collected by the RAND Corporation and are available at http://www.rand.org/labor/FLS/EGSF.html. Women aged 18–35, and up to four of their youngest children, were measured for height and weight. Social network data were also collected providing information on survival status and frequency of contact with grandparents (i.e. the woman's parents and in-laws), as well as details on financial assistance and personal support (advice) the mother received from each grandparent over the past year. Further information was obtained about the child's father's absence from the household as well as demographic information like maternal education, ethnicity, age, the child's birth order and sex. Our models include married women only (i.e. those with in-laws) although almost all sampled women were married (2.3% were not).

Guatemala during the 1990s, and still today, is one of the world's poorest countries and children are severely stunted by World Health Organization (WHO) standards [38]. The total fertility rate in 1995 was 5.24 children per woman and child (under 5) mortality rate was 61 deaths per 1000 live births. The Guatemalan population is divided approximately equally by ethnic identity. Around half are of Mayan, and descendants of other pre-conquest peoples, and the others originate from Spanish and mixed descent, known as Ladina [39]. The ethnic split of the EGSF sample is roughly one-third Ladina, mainly because this is a rural population which accommodates a higher proportion of indigenous people. For both groups, post-marital residence practices are flexible with newlyweds choosing patri-, matri- or neo-local dwellings, as is convenient [40]. The Ladina group tends to own more land, attain professional employment and work in urban centres, while indigenous peoples mainly live on subsistence agriculture and artisan industries, and they are socially and economically marginalized. However, at the time of the EGSF survey, the majority of the inhabitants (i.e. both ethnicities) of rural parts of Guatemala had limited access to clean water, electricity, and public health facilities, and women are poorly educated. The combination of these factors largely explains why Guatemalan children do poorly on world standards of growth [39].

## 3. Methods

The EGSF sample comprises 2892 mothers with 3370 children aged from birth to 5 years. Height for age *z*-scores (HAZ) were calculated based on NCHS/WHO international reference population [41]. To test which grandparents influence child height, we performed multiple regression analyses on a split sample of infants aged 0–12 months and older children aged 1–5 years. Around 17% of mothers had more than one child in the sample and so we included a random effect for mother in the older child models. The infant sample did not include siblings and so no random effect was included in those models. We removed twins from all analyses (*n* = 19). We first examined the relationship between grandparent survival status and contact frequency with mothers, and child height, and then included variables denoting the type of investment grandparents provided—financial and advice. All grandparent variables were categorical. All models controlled for the child's birth order, sex, age and age squared, and the mother's height, age, education, ethnicity, father absence and the department in which the family resides. Distributions of grandparent variables are shown in table 1 and descriptive statistics of all other variables are shown in table 2. Another strength of these data is that there is considerable variation in contact frequency with individual grandparents as well as the types of investment received from each. We, therefore, model grandparental contact and investments individually for each grandparent.

**Table 1.** Distribution of women's interactions with parents and in--laws.

	mother	%	father	%	mother-in-law	%	father-in-law	%
contact frequency								
never	49	1.7	102	3.6	107	4.8	112	5.0
once a month or less	530	18.7	492	17.4	282	12.6	228	10.2
regularly: at least once a fortnight	746	26.4	629	22.3	369	16.5	328	14.7
daily	363	12.8	289	10.2	636	28.5	500	22.4
co-resident	736	26.0	573	20.28	400	17.9	302	13.5
dead	405	14.3	740	26.19	441	19.73	765	34.23
advice (alive grandparents only)								
never	1014	41.8	1225	58.6	1090	60.5	1104	74.7
sometimes	920	37.9	595	28.5	467	25.9	271	18.3
often	493	20.3	269	12.9	246	13.6	103	7.0
financial support (alive grandparents only)								
none	1970	68.8	1459	69.8	1454	80.6	1235	83.6
gave/lent money	757	31.2	630	30.2	349	19.4	243	16.4

**Table 2.** Descriptive statistics for all other variables.

	*n*	%	
time husband spends away			
not resident in household	108	5.9	
often away	193	10.6	
sometimes away	249	13.7	
never away	1268	69.8	
ethnicity			
indigenous	1806	63.3	
Ladina	998	35.0	
other	49	1.7	
department			
Suchitepequez	736	25.9	
Jalapa	755	25.7	
Chimaltenango	737	24.5	
Totonicapan	664	24.0	

	*n*	mean	s.d.
mother's			
height (cm)	2668	147.3	5.8
age	2873	25.8	5.19
highest grades attained	2861	2.7	2.99
children's			
birth order	3370	3.4	1.99
age (months)	3359	33.4	19.08

## 4. Results

**Figure 1.**
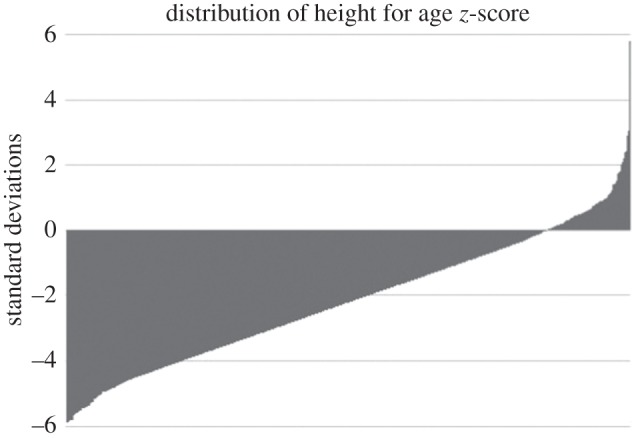
Distribution of HAZ scores in the EGSF sample.

Figure 1 shows the distribution of HAZ scores for this sample of Guatemalan children. It is evident that these children are severely stunted by WHO standards of growth. The mean HAZ score is −2.37 s.d. below the mean; between 2 and −2 is considered healthy by WHO standards [36]. Table 3 shows results from modelling grandparent survival, contact frequency, financial aid and advice received from grandparents, on infant and child height, controlling for father absence and all other demographics. The coefficients represent the number of standard deviations of height for age, above or below the mean of those in the reference category.

**Table 3.** Results of regression analyses for grandparental contact and support on HAZ scores for babies (0–12 months) and 1–5 year old children. matGM, maternal grandmother; patGM, paternal grandmother; matGF, maternal grandfather; patGF, paternal grandfather. All models control for father absence, child's birth order, age, age^2^, sex and mother's height, age, education, ethnicity and regional department (full models can be found in the electronic supplementary material).

	babies (0–12 months)			children (1–5 years)		
height for age *z*-score	model 1 *n*=523 *β* (s.e.)	model 2 *n*=522 *β* (s.e.)	model 3 *n*=522 *β* (s.e.)	model 1 *n*=2448 *β* (s.e.)	model 2 *n*=2443 *β* (s.e.)	model 3 *n*=2443 *β* (s.e.)
contact with matGM (ref: dead)						
never	−0.28 (0.32)	−0.27 (0.32)	−0.27 (0.32)	0.02 (0.19)	0.02 (0.19)	0.01 (0.19)
once a month or less	0.27 (0.18)	0.30 (0.18)	0.33 (0.19)	0.18 (0.08)*	0.19 (0.08)*	0.21 (0.09)*
regularly, at least once a fortnight	0.13 (0.17)	0.20 (0.17)	0.21 (0.18)	0.03 (0.08)	0.03 (0.08)	0.05 (0.09)
daily	0.03 (0.22)	0.14 (0.23)	0.19 (0.24)	0.11 (0.11)	0.12 (0.11)	0.14 (0.11)
co-resident	−0.07 (0.30)	0.01 (0.31)	0.10 (0.33)	−0.11 (0.15)	−0.09 (0.15)	−0.07 (0.16)
contact with patGM (ref: dead)						
never	−0.43 (0.38)	−0.52 (0.39)	−0.52 (0.39)	−0.40 (0.17)*	−0.40 (0.17)*	−0.40 (0.17)*
once a month or less	−0.27 (0.20)	−0.22 (0.20)	−0.16 (0.21)	0.06 (0.10)	0.05 (0.10)	0.03 (0.10)
regularly, at least once a fortnight	−0.38 (0.19)*	−0.37 (0.19)^†^	−0.36 (0.20)	0.03 (0.09)	0.00 (0.09)	−0.04 (0.09)
daily	−0.43 (0.16)**	−0.40 (0.16)*	−0.40 (0.17)*	−0.08 (0.08)	−0.11 (0.08)	−0.14 (0.08)
co-resident	−0.29 (0.19)	−0.26 (0.20)	−0.27 (0.21)	−0.14 (0.10)	−0.17 (0.10)	−0.21 (0.11)
contact with matGF (ref: dead)						
never	0.15 (0.26)	0.09 (0.26)	0.11 (0.26)	0.08 (0.15)	0.05 (0.16)	0.05 (0.16)
once a month or less	−0.07 (0.17)	−0.16 (0.17)	−0.17 (0.18)	−0.04 (0.08)	−0.01 (0.08)	−0.02 (0.08)
regularly, at least once a fortnight	0.05 (0.14)	−0.04 (0.15)	−0.03 (0.16)	0.09 (0.07)	0.13 (0.07)	0.12 (0.08)
daily	0.24 (0.22)	0.11 (0.23)	0.11 (0.23)	−0.08 (0.11)	−0.03 (0.11)	−0.04 (0.11)
co-resident	−0.05 (0.35)	−0.17 (0.36)	−0.19 (0.37)	0.32 (0.18)	0.40 (0.18)*	0.39 (0.18)*
contact with patGF (ref: dead)						
never	−0.34 (0.33)	−0.26 (0.35)	−0.30 (0.35)	−0.01 (0.16)	0.03 (0.16)	0.04 (0.16)
once a month or less	−0.16 (0.20)	−0.12 (0.20)	−0.18 (0.21)	0.12 (0.10)	0.12 (0.10)	0.15 (0.10)
regularly, at least once a fortnight	−0.10 (0.17)	−0.02 (0.18)	−0.03 (0.18)	−0.05 (0.08)	−0.01 (0.09)	0.02 (0.09)
daily	0.01 (0.15)	0.04 (0.16)	0.01 (0.16)	0.08 (0.07)	0.11 (0.08)	0.13 (0.08)
co-resident	−0.28 (0.20)	−0.25 (0.20)	−0.31 (0.21)	0.06 (0.11)	0.08 (0.11)	0.11 (0.11)
money from matGM (ref: none)						
gave and/or lent money		−0.24 (0.14)	−0.23 (0.15)		−0.02 (0.07)	−0.01 (0.08)
money from patGM (ref: none)						
gave and/or lent money		−0.08 (0.16)	−0.05 (0.16)		0.16 (0.08)*	0.14 (0.08)
money from matGF (ref: none)						
gave and/or lent money		0.34 (0.16)*	0.34 (0.16)*		−0.20 (0.08)*	−0.21 (0.09)*
money from patGF (ref: none)						
gave and/or lent money		−0.18 (0.19)	−0.22 (0.19)		−0.13 (0.09)	−0.09 (0.09)
advice from matGM (ref: never)						
sometimes			0.01 (0.15)			−0.05 (0.07)
often			−0.24 (0.20)			0.00 (0.10)
advice from patGM (ref: never)						
sometimes			−0.14 (0.14)			0.05 (0.07)
often			0.10 (0.20)			0.13 (0.10)
advice from matGF (ref: never)						
sometimes			0.02 (0.16)			0.04 (0.08)
often			−0.10 (0.26)			0.00 (0.13)
advice from patGF (ref: never)						
sometimes			0.04 (0.18)			−0.09 (0.09)
often			0.30 (0.29)			−0.18 (0.14)
intercept	−7.99	−7.99	−8.08	−14.43	−14.43	−14.43

*p < 0.05, **p < 0.01, ^†^*p* = 0.051.

**Figure 2.**
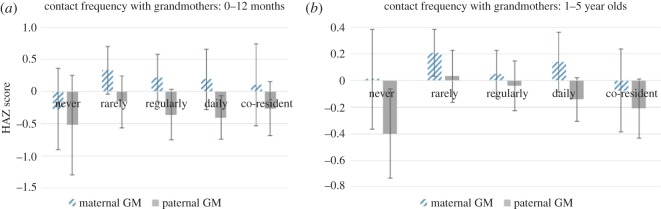
HAZ scores for each level of contact with grandmothers, adjusted for birth order, child age, child age squared, sex, mother's education, age, height, ethnicity, father absence and department. (*a*) Infants; (*b*) 1–5 year olds.

*Contact frequency*. For babies, 12 months and younger, we find evidence for a relationship between contact frequency with paternal grandmothers and infant length: regular and daily contacts are both associated with shorter babies. For children aged 1–5 years, effect sizes are smaller suggesting grandparental influence may be weaker in older children, but the relationship between paternal grandmother contact and child height is still largely negative. By contrast, maternal grandmothers are broadly positively associated with infant length and child height: coefficients are largely positive, and occasionally seeing a maternal grandmother is significantly associated with taller stature in the 1–5 age group. Figure 2*a,b* illustrates the impact of each category of maternal and paternal grandmother contact on (i) infant length and (ii) child height, clearly demonstrating the opposite effects of maternal and paternal grandmothers. There is weak evidence that having a co-resident maternal grandfather may also improve child height, as co-residence with maternal grandfather is significantly associated with height in older children. Other kinds of contact with maternal grandfathers are not consistently or significantly associated with infant or child height. Paternal grandfathers too are not consistently or significantly associated with child height in either age group.

*Financial assistance*. The relationship between financial assistance from grandparents and child height is more complicated. When adding variables for financial assistance in the models, only financial assistance from maternal grandfather is significantly associated with child height in all models; however, this relationship is positive for infants, but negative for older children.

*Advice*. We found no evidence that the provision of advice from grandparents made any difference to child height: point estimates are close to zero, none are significant, and the signs of coefficients show no particular patterns.

**Figure 3.**
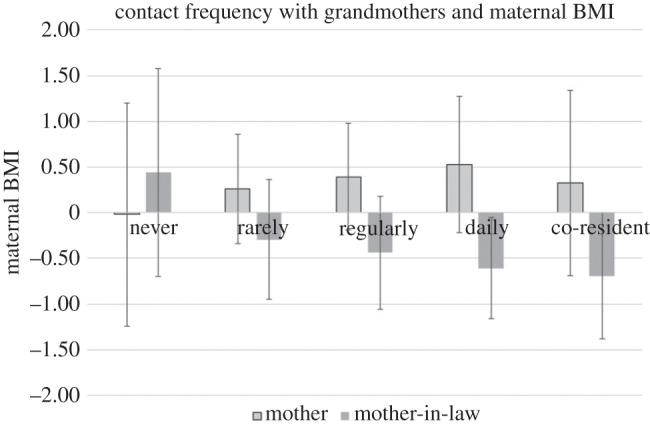
Maternal BMI scores for each level of contact with grandmothers, adjusted for age, education, ethnicity, husband absence and department.

In sum, the clearest evidence we have for grandparental influences suggests that contact with paternal grandmothers is associated with shorter child height. We further find some evidence that contact with maternal grandparents, particularly grandmothers, may be beneficial for child height. It is possible that grandparental influences partly act through maternal health, particularly as the effect sizes for these associations are larger for babies than for older children. We performed multivariate linear regression analyses to test if grandparental contact (and other forms of investment) was associated with maternal body mass index (BMI). Patterns of associations between contact with grandmothers and maternal BMI broadly show the same results as for child height. Contact, particularly frequent contact, with paternal grandmothers is associated with significantly lower maternal BMI. Contact with maternal grandmothers is not significantly associated with maternal BMI, although the direction of associations is similar to those for child height (figure 3). We found little evidence for an effect of grandparent advice and financial assistance on maternal BMI.

## 5. Discussion

Overall, we see a positive impact of maternal grandmothers' contact with children on child height. These results are particularly apparent for infants. By and large the impact of contact with grandfathers on child health is limited. This population practices flexible post-marital residence where some newlyweds live with the husband's family after marriage but if that is inconvenient, they may live with the wife's family or they may live in a separate dwelling, close to either the parents or in-laws [40]. The patterns of contact frequency in our sample reveal this pattern too: women tend to have slightly more contact with their in-laws although many women see their own parents regularly and even daily. Previous research suggests that maternal grandmothers might typically be more beneficial for child health and survival [6,7,12,42], except in matrilineal populations where members of the matriline compete for limited resources [9]. Strassmann & Garrard [7] suggest that resource competition is the explanation for the typically less beneficial effects of paternal grandmothers compared with maternal grandmothers, given that most human populations are patrilineal. Our findings lend support to this resource competition hypothesis. Not only were children shorter in the presence of paternal grandmothers, but women had lower BMIs if they were in frequent contact with their mothers-in-law. These findings also fit with Beise & Voland's [33] suggestion that conflict between women and their mothers-in-law may play a role, as the effects were particularly notable in infancy. The slightly better child health seen with contact with maternal grandmothers, perhaps supported by higher maternal BMI, suggests that contact with maternal grandmothers may involve helping behaviour between mothers and daughters.

The more detailed data we have used on financial assistance and advice given to women by their kin have not helped shed much light on how family support might influence child health. There was no evidence that advice given to women made any difference to children's, or women's, health. Previous research in other populations has suggested that older women do have important roles in giving advice around childbirth and childcare to women; however, quite sizeable proportions of women claimed never to receive advice from grandparents in this population, although this may reflect difficulties in interpreting the question. We also have no information about the content or quality of advice given.

We find evidence for an association between maternal grandfather financial support and child height but the estimates are in opposite directions for babies (positive) and older children (negative). One problem with interpreting data on financial assistance from relatives is that the provision of financial support may be based on the recipients' need. Snopkowski & Sear [43], for example, found that poorer families received preferential financial aid from grandparents (also see Schaffnit & Sear [44] for negative effects of financial support on fertility). Receiving financial aid, therefore, may be an indication either that a family is particularly in need of help (which may lead to a negative association between the receipt of financial support and child health), or that a family is receiving extra resources from relatives, which can be used to boost child health (leading to a positive association between financial support and health). To test whether financial support might be an indication of need in our sample, we examined what might predict financial support from grandparents. We found that women with absent husbands were more likely to receive financial support from their parents but not their in-laws, suggesting that financial input from the woman's own family may be at least partly needs-based, although other factors also accounted for some of the variations in financial support (such as education and ethnicity). We unfortunately have little information about these absent husbands, and absent husbands could result from labour migration, in which case they may still be contributing financially to a household even in their absence. Overall then, our results suggest that there is considerable complexity within family relationships and their influence on child health: both competition and cooperation within the family may explain grandparental influences on child health, but results may also be confounded by differential treatment of children and grandchildren by grandparents, if grandparents direct some kinds of support preferentially to those children and grandchildren most in need.

## 6. Limitations

As with all cross-sectional studies we can only infer effects from correlations and not assume that these relationships indicate a causal pathway. We also acknowledge that in observational studies, there could be confounding factors that we cannot account for. We have, nevertheless, controlled for as many potential confounders as possible. Also, it should be noted that these data were not collected with our research question specifically in mind so we are limited by the data available. We have tested a large number of variables, raising the possibility that some significant results may be spurious (5% of the time seemingly significant effects are random). However, we have based our interpretations on the pattern of our results, shown by the direction of coefficients, as well as statistical significance.

## 7. Conclusion

We used a rich dataset from rural Guatemala to model the individual effects of grandparental presence, contact frequency and direct investments (advice and financial) on child height. Overall, our results support previous research finding that maternal grandmothers tend to be beneficial for their grandchildren, although this relationship is weak and many coefficients, although in the positive direction, are non-significant. We find a negative relationship between contact with paternal grandmothers and child height, supporting the hypothesis that paternal grandmothers and grandchildren compete for resources within patrilines. Previous work correlating the survival status of grandparents with the survival of grandchildren has been criticized for not being able to exclude the possibility that these relationships are driven by the influence of shared genes or environments within families, i.e. families with long-lived grandparents might be healthy or wealthy families who are also able to ensure the survival of young children. Our analysis does not suffer from such problems. We should point out that there were no significant findings for paternal grandfathers, nor for advice from any grandparent. The overall picture gleaned from these data, therefore, may suggest that grandparents overall have a relatively small impact on child height in this population. However, Guatemalan children fare very poorly by world standards of nutrition meaning that even small amounts of support (or non-support) from grandparents can have a meaningful impact on child health.

## Supplementary Material

Full information for all models.
